# DMSC: A Dynamic Multi-Seeds Method for Clustering 16S rRNA Sequences Into OTUs

**DOI:** 10.3389/fmicb.2019.00428

**Published:** 2019-03-12

**Authors:** Ze-Gang Wei, Shao-Wu Zhang

**Affiliations:** ^1^Key Laboratory of Information Fusion Technology of Ministry of Education, School of Automation, Northwestern Polytechnical University, Xi’an, China; ^2^Institute of Physics and Optoelectronics Technology, Baoji University of Arts and Science, Baoji, China

**Keywords:** multi-seeds, dynamic update, clustering, operational taxonomic units, 16S rRNA

## Abstract

Next-generation sequencing (NGS)-based 16S rRNA sequencing by jointly using the PCR amplification and NGS technology is a cost-effective technique, which has been successfully used to study the phylogeny and taxonomy of samples from complex microbiomes or environments. Clustering 16S rRNA sequences into operational taxonomic units (OTUs) is often the first step for many downstream analyses. Heuristic clustering is one of the most widely employed approaches for generating OTUs. However, most heuristic OTUs clustering methods just select one single seed sequence to represent each cluster, resulting in their outcomes suffer from either overestimation of OTUs number or sensitivity to sequencing errors. In this paper, we present a novel dynamic multi-seeds clustering method (namely DMSC) to pick OTUs. DMSC first heuristically generates clusters according to the distance threshold. When the size of a cluster reaches the pre-defined minimum size, then DMSC selects the multi-core sequences (MCS) as the seeds that are defined as the *n*-core sequences (*n* ≥ 3), in which the distance between any two sequences is less than the distance threshold. A new sequence is assigned to the corresponding cluster depending on the average distance to MCS and the distance standard deviation within the MCS. If a new sequence is added to the cluster, dynamically update the MCS until no sequence is merged into the cluster. The new method DMSC was tested on several simulated and real-life sequence datasets and also compared with the traditional heuristic methods such as CD-HIT, UCLUST, and DBH. Experimental results in terms of the inferred OTUs number, normalized mutual information (NMI) and Matthew correlation coefficient (MCC) metrics demonstrate that DMSC can produce higher quality clusters with low memory usage and reduce OTU overestimation. Additionally, DMSC is also robust to the sequencing errors. The DMSC software can be freely downloaded from https://github.com/NWPU-903PR/DMSC.

## Introduction

Bacteria are the most diverse domain on our planet and play an essential role in various biogeochemical activities as well as an important role in human health and disease (Fuks et al., 2018). Characterizing the taxonomic community composition taken from an environmental sample is critical for understanding the bacterial world (Lane et al., 1985; Wei et al., 2016). Most of our knowledge about the microbial community descriptions comes from the 16S rRNA (ribosomal RNA) marker genes generated by high-throughput sequencing technology (Koslicki et al., 2013). Bypassing the necessity of isolating single organisms for cultivation, the advanced sequencing technology can produce millions of 16S rRNA and has become a powerful tool for in-depth analysis of bacterial community composition (Zhang et al., 2013; Wei and Zhang, 2018).

Usually, a fundamental first step for rapidly processing the 16S sequencing data is to cluster them into the OTUs (Turnbaugh et al., 2007; Peterson et al., 2009), which form the basis for estimating the species, diversity, composition, and richness of the microbes in the environment (Amir et al., 2017; Westcott and Schloss, 2017). Two major approaches for binning 16S rRNA sequences include: (i) taxonomy dependent methods, where each query sequence is compared against a reference taxonomy database and assigned to the organism of the best-matched annotated sequence using sequence searching (Altschul et al., 1990) or classification (Liu et al., 2017, 2018), and (ii) taxonomy independent methods (also called *de novo* clustering) (Chen et al., 2013b), where sequences are grouped into OTUs based on pairwise sequence similarities. However, a significant portion of microbes in a sample is contributed by unknown taxa which are not recorded in databases, thus taxonomy dependent methods are inherently limited by the completeness of reference databases (Chen et al., 2016). In contrast, *de novo* clustering methods divide sequences into OTUs without needing any reference database and have become the preferred choice for researchers (Cai et al., 2017).

In the past decade, a wide variety of *de novo* clustering methods has been proposed for binning OTUs. These methods can be further categorized into hierarchical clustering, heuristic clustering, model-based and network-based methods (Wei et al., 2017). Hierarchical clustering methods [e.g., mothur (Schloss et al., 2009), HPC-CLUST (Matias Rodrigues and von Mering, 2013), ESPRIT (Sun et al., 2009), and mcClust (Cole et al., 2013)] require a distance matrix derived either from all pairs sequences alignment or a multiple sequence alignment, then build a hierarchical tree with a predefined threshold to assign sequences into OTUs. Network-based methods [e.g., M-pick (Wang et al., 2013) and DMclust (Wei et al., 2017)] first construct a fully connected graph by computing all pairwise sequences distances and then employ the strategy of modularity community detection to generate OTUs. As a result, the computational complexity of both hierarchical and network-based methods is *O*(*N^2^*), where *N* is the number of sequences (Wei and Zhang, 2017; Wei et al., 2017). Model-based methods [e.g., CROP (Hao et al., 2011) and BEBaC (Cheng et al., 2012)] mainly apply some statistical model (e.g., Bayesian model) or mathematics framework (e.g., Gaussian mixture model) to describe sequence data then assign sequences to OTUs based on probability theory, and still, have a high computational burden (Chen et al., 2013a). Therefore, hierarchical clustering, model-based and network-based clustering methods quickly meet with the bottleneck in terms of computational time and memory usage for dealing with large-scale sequencing data (Wei et al., 2017).

A dozen of heuristic clustering methods such as CD-HIT (Li and Godzik, 2006), UCLUST (Edgar, 2010), DySC (Zheng et al., 2012), VSEARCH (Rognes et al., 2016), and DBH (Wei and Zhang, 2017) were developed to decrease the computational complexity. These methods build up clusters in an iterative incremental strategy. Each cluster is represented by one sequence (called seed) and each sequence is compared to all seeds. If the distance between one input sequence and a seed is within a given threshold, the input sequence is assigned to an existing cluster. Otherwise, this sequence becomes a seed of a new cluster. This procedure is repeated until all sequences are assigned. The computational complexity of heuristic clustering methods is *O*(*NM*), where *M* is the number of seeds (usually *M* ≤*N*). Therefore, heuristic clustering methods run several orders of magnitude faster than other clustering algorithms and are more widely used in processing millions of 16S rRNA sequences (Cai and Sun, 2011).

Although heuristic clustering approaches are computationally efficient, they always overestimate the OTUs number and produce lower clustering quality than other methods (Huse et al., 2010; Wei and Zhang, 2015). Because most existing heuristic clustering methods just use one single sequence as the seed for each cluster, the results show an obvious sensitivity to the selected seeds that represent the clusters, especially when sequences datasets contain sequencing errors (Zheng et al., 2012; Chen et al., 2013a; Wei and Zhang, 2017). Therefore, selecting “good” seeds for one cluster is profoundly significant for heuristic clustering methods. In this work, inspired by the seed reselection procedure in DySC and the Gaussian model representation of one cluster in CROP, we proposed a **d**ynamic **m**ulti-**s**eeds **c**lustering (namely DMSC) method to pick OTUs. The DMSC algorithm consists of four main phases. First, heuristically generate clusters according to the distance threshold, which is similar to classical heuristic methods (e.g., CD-HIT or UCLUST). Second, when the size of a cluster reaches the pre-defined minimum size, select the MCS as seeds of a cluster, in which the distance between any two sequences is less than the distance threshold. Third, a new sequence is assigned to the corresponding cluster depending on the average distance to MCS and the distance standard deviation between each pairwise sequences in MCS. Finally, DMSC dynamically updates the MCS until no sequence is merged into the cluster.

Compared with other heuristic clustering methods, the unique characteristics of our DMSC method mainly manifest in the following three points. (i) DMSC selects MCS as the seeds in one cluster instead of the single seed representation used in most heuristic clustering methods such as CD-HIT and UCLUST; (ii) in DMSC, the MCS of one cluster is always dynamically updated with the cluster size increases, while the seed of each cluster in most other heuristic methods is always fixed; and (iii) according to the average distance to MCS and the distance standard deviation between each pairwise sequences in MCS, a new sequence is assigned to the corresponding cluster, while other heuristic methods assign the new sequence to one cluster just base on the distance with the seed sequence. Four experimental results demonstrate that DMSC can achieve higher quality clusters and reduce OTU overestimation with low memory usage. Additionally, DMSC is also robust to sequencing errors.

## Materials and Methods

**FIGURE 1:**
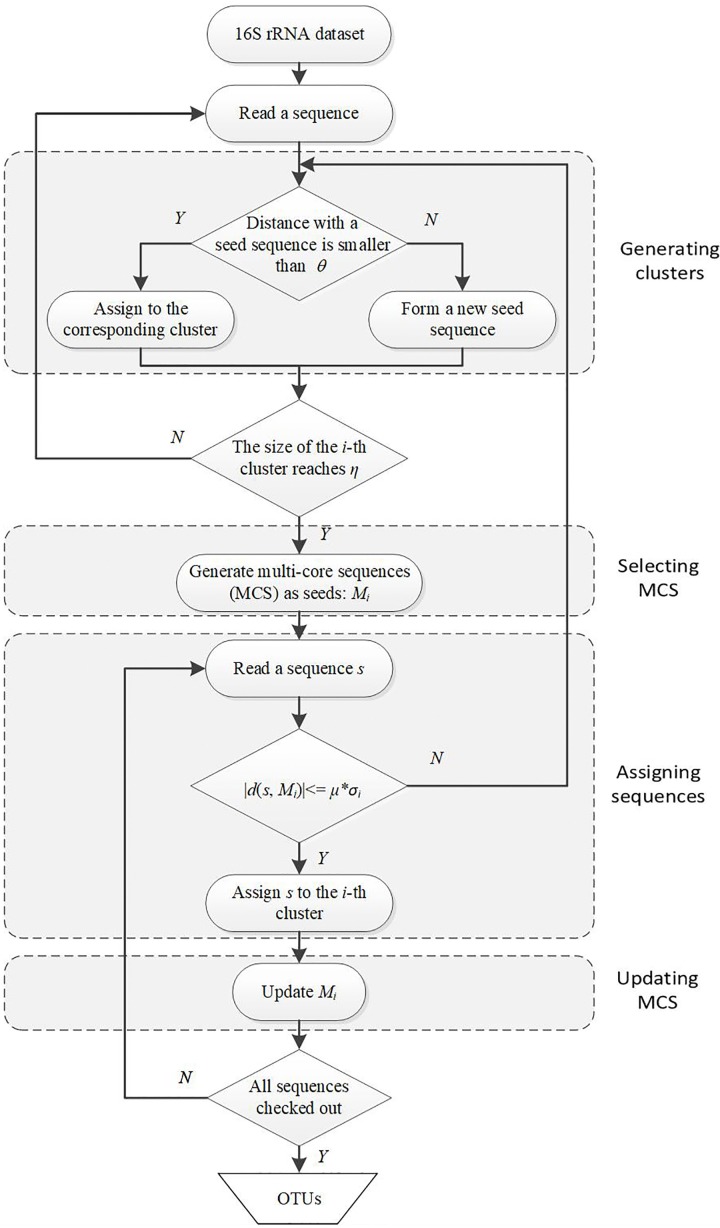
Flowchart of DMSC algorithm. DMSC contains four main modules: generating clusters, selecting MCS, assigning sequences, and updating MCS. *θ* denotes the distance threshold; η is the minimum sequence number of a cluster to select the MCS; *M_i_* is the MCS of the i-th cluster; σ*_i_* is the distance standard deviation between each pairwise sequences in *M_i_*; and μ denotes the multiple.

The first motivation of our DMSC method is to decrease the sensitivity of single seed representation to sequencing errors in most heuristic clustering methods. Here we select the MCS as seeds of a cluster, in which the distance between any two sequences is less than the distance threshold. There are two different parameters in DMSC approach: η (default value 25), the minimum sequence number in a cluster to ensure that the cluster contains enough sequences to yield a reliable MCS; and μ (default value 3), the time (multiple) of distance standard deviation between each pair of sequences in the MCS. These parameter settings have been evaluated in following experiments and the default values have robust performance. Figure 1 is a flowchart showing the OTUs generating process with DMSC. It can be seen that DMSC method has four main phases: (i) according to the distance threshold *θ*, a series of clusters are formed by heuristic clustering of each sequence one by one; (ii) when the size of a cluster reaches the pre-defined minimum sequence number (η), the MCS is selected as the seeds; (iii) according to the average distance to MCS and the distance standard deviation (σ) between each pairwise sequences in MCS, a new sequence is assigned to the corresponding cluster; and (iv) after a new sequence is added to one cluster, update the MCS.

### Generating Clusters

At the beginning of DMSC, the input sequences are sorted by abundance in a descending order. These can eliminate the influence of sequence input order on the clustering results. Then the first sequence is assigned to the first cluster and becomes the seed of this cluster. The second sequence is added to the cluster if the distance between the sequence and the seed is within the pre-defined threshold (*θ*), otherwise, this sequence is stored as a new seed for creating a new cluster. Repeat this process until the size of a cluster reaches the predefined threshold (η), then the MCS selection procedure is activated.

### Selecting Multi-Core Sequences (MCS)

**FIGURE 2:**
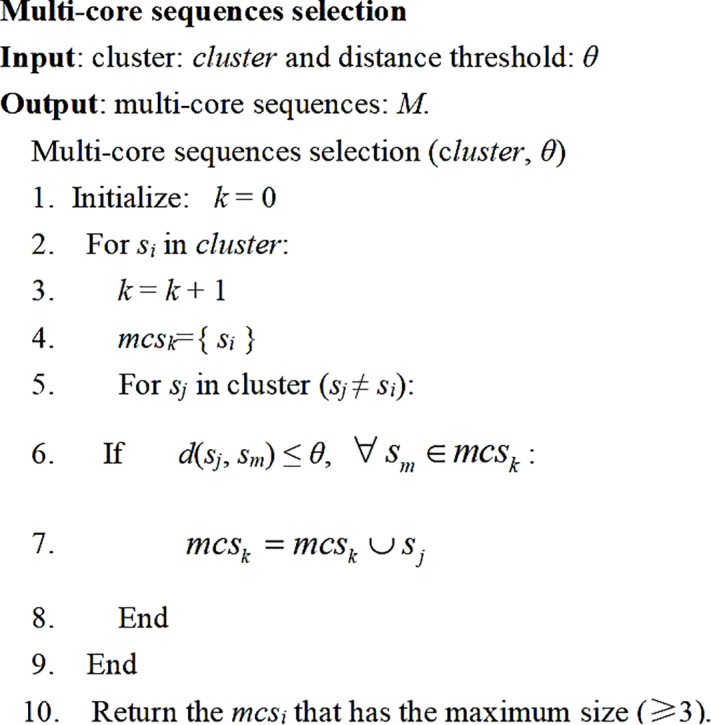
The pseudo-code of the MCS selection procedure for one cluster.

The multi-core sequences of one cluster is defined as the *n*-core sequences (*n* ≥ 3), in which the distance between any two sequences within the cluster is less than the distance threshold (*θ*). If more than 3-core sequences are selected in the cluster, these core sequences are taken as seeds to represent this cluster, otherwise, one seed sequence is selected to represent this cluster. Although the MCS selection procedure can reduce OTU overestimation and decrease the sensitivity to the sequencing errors, it will increase the computational burden. Considering both the clustering quality and the computational burden, we select more than 3 core sequences (i.e., *n* ≥ 3) as the seeds in this paper. The pseudo-code for the MCS selection procedure is outlined in the following Figure 2.

### Assigning Sequences

One reason that heuristic clustering methods generally overestimate the OTUs number is that these methods just compare the distance with single seed to assign sequences. Model-based clustering methods can reduce OTU overestimation because they consider the distance distribution in one cluster. Therefore, we introduce the distance standard deviation (σ) between each pairwise sequences in one MCS in this work. That is:

(1) $$|d\left(s,M_{i}\right)|\leq \mu *\sigma_{i}$$

where *M_i_* is the MCS of the *i*-th cluster, *d*(*s*, *M_i_*) is the average distance between sequence *s* and *M_i_*, μ is the multiple constant, σ*_i_* is the distance standard deviation of *M_i_*. If the sequence *s* meets Equation 1, then *s* is merged into the *i*-th cluster. *d*(*s*, *M_i_*) and σ*_i_* are defined as:

(2) $$d\left(s,M_{i}\right)=\frac{1}{\left|M_{i}\right|}\overset{\left|M_{i}\right|}{\underset{i=1}{\Sigma }}d(s,s_{i}),s_{i}\in M_{i}$$

(3) $$\sigma_{i}=\sqrt{\frac{1}{|M_{i}|-1}\overset{s_{i}\neq s_{j}}{\underset{s_{i},s_{j}\in M_{i}}{\Sigma }}{\left[d(s_{i},s_{j})-{\overset{¯}{d}}_{M_{i}}\right]}^{2}}$$

where |*M_i_*| is the sequence number in *M_i_*, ${\overset{¯}{d}}_{M_{i}}$ is the average distance of all pairwise sequences in *M_i_*.

### Updating MCS

Once one sequence is merged into a cluster, the MCS will be updated according to the MCS selection procedure in Figure 2. Therefore, the MCS of one cluster is always dynamically updating with the cluster size increases.

After all the MCSs are no long change, all the isolated sequences are checked and assigned to the nearest neighbor clusters to form OTUs.

## Results

We compared our DMSC method with seven state-of-the-art OTUs clustering algorithms: CD-HIT (v.4.6.8) (Li and Godzik, 2006), UCLUST (v.11.0.667) (Edgar, 2010), DBH (Wei and Zhang, 2017), DySC (Zheng et al., 2012), ESPRIT-Forest (Cai et al., 2017), AL clustering algorithm implemented in mothur (v.1.40.5) (Schloss et al., 2009), and CROP (Hao et al., 2011). Among these methods, CD-HIT, UCLUST, DySC, and DBH are typical heuristic clustering approaches; mothur is an open source software package for analyzing the biological sequence data, and the AL clustering in mothur (mothur-AL) has been demonstrated that it is a reliable method to represent the actual distances between sequences (Westcott and Schloss, 2015); ESPRIT-Forest is a new parallel hierarchical clustering method, and CROP is a model-based method. We conducted these methods on four benchmark datasets including two simulated dataset and three published real-life datasets. Some features of each benchmark dataset are shown in Table 1.

**Table 1:** Details of the benchmark datasets.

Datasets	Taxon number	Sequence number	Average length	Variable regions	Data source
Stacked_60 dataset	59	2,614	98 bp	V6	Barriuso et al., 2011
Simulated dataset	11	22,000	500 bp	–	Cheng et al., 2012
V6 dataset	177	∼310 K	121 bp	V6	Chen et al., 2013a
V4 dataset	68	∼511 K	253 bp	V4	Westcott and Schloss, 2015
Error datasets	30	150 K	120 bp	V6	Wei and Zhang, 2017


The metrics of OTUs number, NMI, and MCC are adopted to access the performance of every OTU picking method in the following experiments. The metrics of OTUs number and NMI have been widely used to compare the performance of OTU picking methods based on the known ground truth information datasets (Sun et al., 2011; Schmidt et al., 2015). Although the ground truth information (i.e., how many species the dataset includes, and what species the sequence belongs to) is always unknown for most real-life 16S rRNA sequencing dataset, it can be partially resolved by applying some searching methods against the reference database to annotate the 16S rRNA sequences (Cai and Sun, 2011; Chen et al., 2013b; Edgar, 2018). MCC metric was also used to evaluate the performance of OTU picking methods based on the sequence distance and clustering threshold without relying on an external reference (Schloss and Westcott, 2011), which is an objective metric to assess the clustering quality of OTUs picking methods (He et al., 2015; Westcott and Schloss, 2015; Schloss, 2016). The computational formulas of NMI and MCC are listed in Supplementary File.

All methods were executed on an Ubuntu 16.04.5 server with 16 3.2-GHz Intel Xeon (E5-2667V4) processors and 128 GB of RAM. And the running command lines of each method are listed in Supplementary Table S1.

### Experiment 1: Stacked_60 Dataset

The Stacked_60 benchmark dataset was constructed by Barriuso et al. (2011), which is retrieved from 59 different bacterial genera in the NCBI and trimmed to obtain the V6 region (from positions 963 to 1063 in *E. coli*). Stacked_60 contains random mutation and is specially designed to test the accuracy of OTUs picking methods at different sequence distances. The taxa distance range and the taxa abundance are in 0.01–0.38 and 0.001–0.003, respectively.

Table 2 lists the maximum NMI value and the corresponding OTUs number, from which we can see that DMSC and CROP have higher maximum NMI value than the other methods, and different methods achieve the maximum NMI values at different distance thresholds. At the respective maximum NMI value, DMSC and CROP inferred 59 OTUs which equals to the expected number, while DBH, DySC, CD-HIT, mothur-AL and ESPRIT-Forest overestimated OTUs number, and UCLUST underestimated OTUs number.

**Table 2:** The maximum NMI values and OTUs number with different methods on stacked_60 dataset.

Methods	DMSC (0.03)	CROP (0.03)	DySC (0.03)	DBH (0.02)	CD-HIT (0.04)	UCLUST (0.09)	mothur-AL (0.06)	ESPRIT-Forest (0.08)
Max. NMI	0.99951	0.99951	0.99475	0.99868	0.99557	0.98528	0.96650	0.96614
OTUs	59	59	60	62	65	56	161	86


*The value in the bracket is the distance threshold where each method achieves its maximum NMI. For mothur-AL method, the maximum NMI of mothur-AL is selected from the distance range of 0.01∼0.06 for reason that mothur-AL method just obtains the clustering results in these distance thresholds.*

**FIGURE 3:**
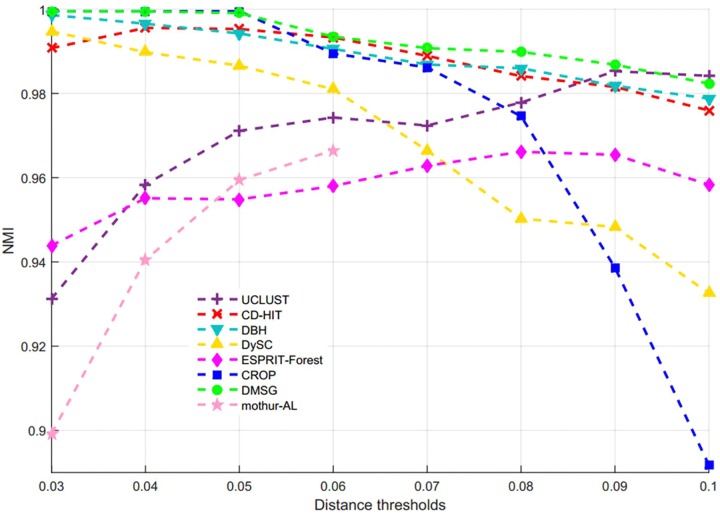
NMI values of different algorithms on stacked_60 dataset.

**FIGURE 4:**
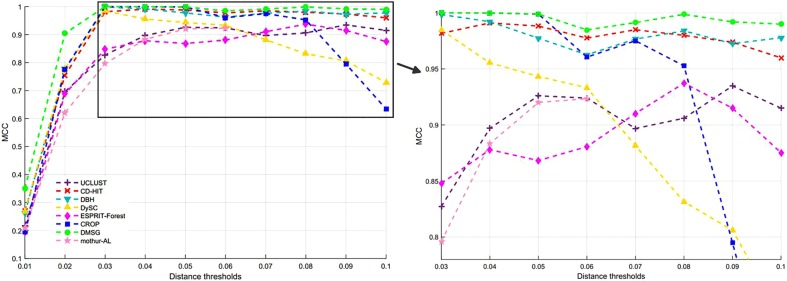
MCC values of different methods on stacked_60 dataset.

Figure 3 shows the NMI values of DMSC, CROP, UCLUST, CD-HIT, DySC, DBH, mothur-AL, and ESPRIT-Forest with different distance thresholds on the Stacked_60 dataset. It can be seen that the NMI value of DMSC is almost identical to the CROP from 0.03 to 0.05 distance threshold, and also higher than that of other methods. In the range of 0.06∼0.09, DMSC achieved the highest NMI values, while the NMI value of CROP continuously drops, indicating that CROP is more sensitive to the distance threshold. Because the NMI values vary a lot in the range of 0.01∼0.02 distance thresholds for all methods, Figure 3 just represents the NMI values from 0.03 to 0.10 distance thresholds. Figure 4 depicts the MCC curves of eight methods with different distance thresholds on Stacked_60 dataset. From Figure 4 we can see that DMSC method always achieved the highest MCC value in the range of 0.01∼0.10 distance thresholds. The NMI values, OTUs number and MCC values of eight methods in the range of 0.01∼0.1 distance thresholds can be found in Supplementary Table S2.

These results in Figure 3, 4, Table 1, and Supplementary Table S2 show that our DMSC method can accurately estimate the species number and obtain better cluster quality for Stacked_60 dataset.

### Experiment 2: Simulated Dataset

We then considered another widely used simulated dataset to estimate the clustering accuracy, where the ground truths were directly taken from a simulator software (Cheng et al., 2012). A total of 22,000 sequences (∼500 bp) from 11 taxa were generated and each taxon contains 2,000 sequences with different substitution rates. Among these 11 taxa, three taxa are within 1% different from each other. Therefore, the expected OTUs number is 9.

**FIGURE 5:**
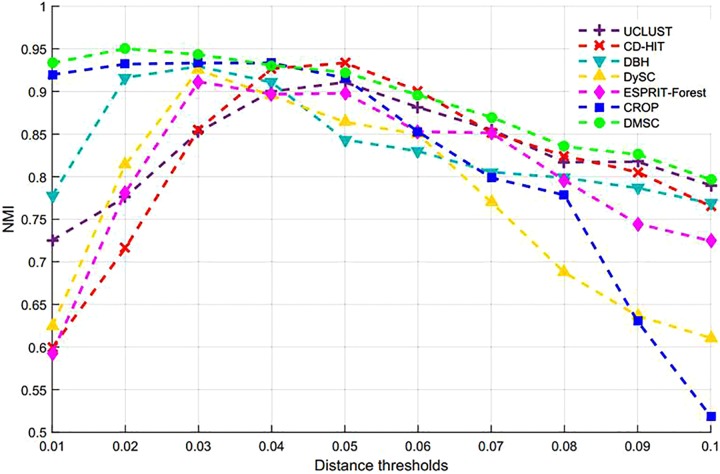
NMI values of different methods on the simulated dataset.

**FIGURE 6:**
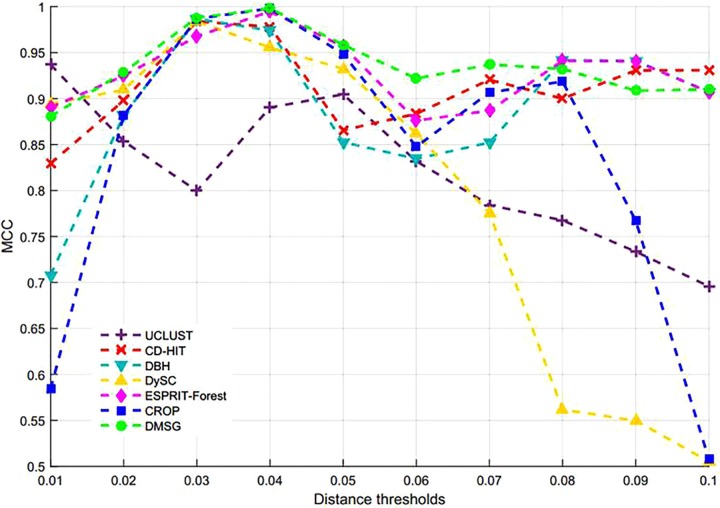
MCC values of different methods on the simulated dataset.

By setting different distance thresholds ranging from 0.01 to 0.1, the maximum NMI values of seven methods at different distance thresholds and the corresponding inferred OTUs number are reported in Table 3, from which we can see that DMSC achieved the highest NMI (0.9503). Meanwhile, DMSC, CROP, DBH, and CD-HIT successfully obtained 9 OTUs at their best NMI value, while DySC, UCLUST, and ESPRIT-Forest overestimated OTUs. The NMI curves of seven methods are shown in Figure 5, from which we can see that DMSC achieved better NMI values than other methods at distance intervals [0.01, 0.04] and [0.07, 0.1], reaching the highest NMI value at 0.02 distance threshold; other methods obtained their best NMI values at different distance thresholds. Figure 6 represents the MCC curve of seven methods with different distance thresholds ranging from 0.01 to 0.1, from which we can see that MCC values of DMSC are higher than that of other six methods in the range of 0.02∼0.07 distance thresholds. The NMI values, OTUs number and MCC values of seven methods are listed in Supplementary Table S3. These results indicate that DMSC has a better cluster performance than ESPRIT-Tree, CD-HIT, UCLUST, DBH, CROP, and DySC.

**Table 3:** Maximum NMI values of seven methods on the simulated dataset.

Methods	DMSC (0.02)	CROP (0.03)	DySC (0.03)	DBH (0.03)	CD-HIT (0.05)	UCLUST (0.05)	ESPRIT-Forest (0.05)
Maximum NMI	0.9503	0.9334	0.9252	0.9293	0.9334	0.9107	0.8979
OTUs number	9	9	17	9	9	10	13


*The value in the bracket is the distance threshold where each method achieves its maximum NMI.*

### Experiment 3: V6 Variable Region Dataset From Human Gut Flora

In this experiment, we use one real-world benchmark dataset of the V6 variable region from human gut flora to evaluate the performance of OTUs picking methods. This dataset contains ∼310K sequences (average length: ∼121 bp) which are classified into 177 species and covers the V6 hypervariable region of 16S rRNA gene (Chen et al., 2013a). In order to reduce computational burden and remove statistical variations, each method was run 10 times and ∼30K reads were randomly extracted from the V6 dataset in each run.

**FIGURE 7:**
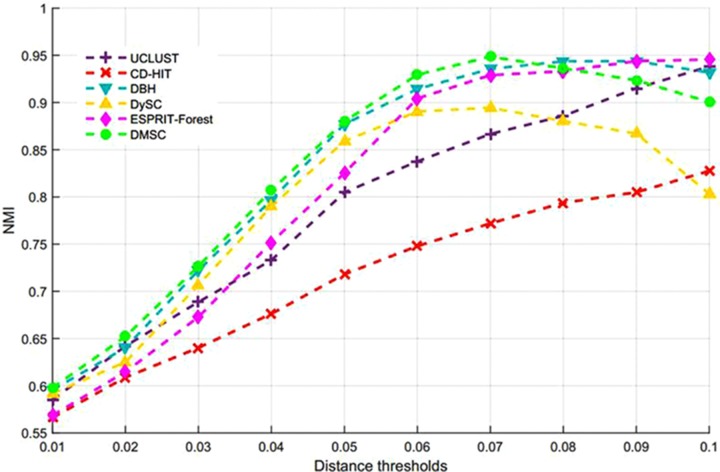
Average NMI values of six methods at different distance thresholds on V6 data set.

**FIGURE 8:**
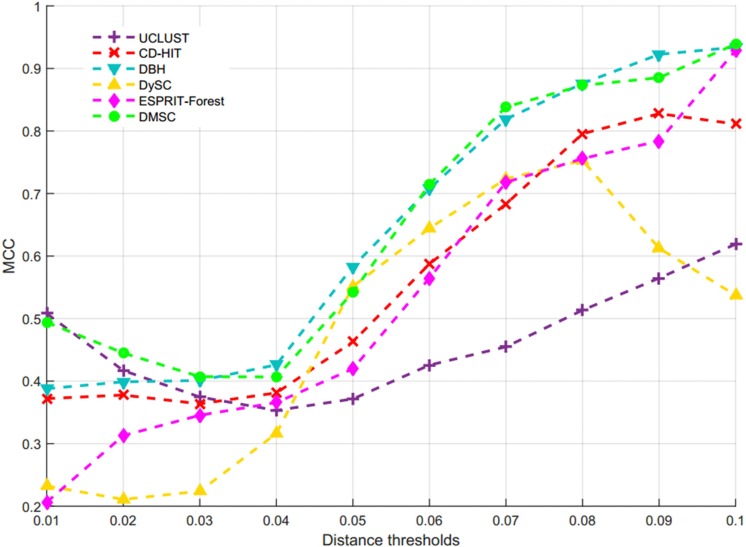
The MCC values of six methods on V6 dataset.

Figure 7 describes the average NMI value as a function of the distance threshold over 10 runs for six methods, from which we can observe that DMSC has the highest NMI values than other methods in the range of 0.01∼0.07 distance thresholds, and DBH also achieved higher NMI values than CD-HIT, UCLUST, DySC, and ESPRIT-Forest from distance threshold interval [0.02, 0.08]. CD-HIT has the lowest NMI values except at 0.1 distance threshold. The average OTUs number inferred with six methods at different distance thresholds are described in Supplementary Figure S1, from which we can see that DMSC inferred fewer OTUs than CD-HIT, UCLUST, DBH and ESPRIT-Forest, but more than DySC at different distance thresholds. These can be explained by the fact that the sequence distance calculation in DySC is based on pairwise *k*-mer distances (Zheng et al., 2012), while other methods (including DMSC) are based on pairwise sequence alignment (PSA). It’s reported that *k*-mer distance is looser than PSA (Sun et al., 2009). In other words, when setting to the same threshold (e.g., 0.03), more sequences of using the *k*-mer distance will satisfy the threshold to be clustered into one group, resulting in that DySC trends to generate fewer OTUs. However, DySC always gives less clustering accuracy and quality than DMSC in terms of the NMI (Figure 7) and MCC (Figure 8) evaluation metrics. Supplementary Figure S2 reports the NMI std of six methods at different distance thresholds with 10 re-sampled runs, from which we can see that the NMI std of DMSC varies in the scope of 0.003∼0.012 at different distance thresholds. DMSC has the lowest std than other five methods in the range of 0.06∼0.09 distance thresholds and almost equals to CD-HIT and UCLUST in the range of 0.01∼0.05 distance thresholds. Figure 8 presents the MCC curves of six methods with different distance thresholds, from which we can see that the MCC values of DMSC and DBH are higher than that of other four methods in the range of 0.03∼0.10 distance thresholds. For reason that CROP takes longer running time to output the OTUs for the large-scale dataset, we did not list the results of CROP in this experiment. Supplementary Table S4 lists the NMI values, OTUs number and MCC values of six methods, and Supplementary Table S5 gives the *t*-test results of DMSC compared with the other four methods. These results in Figure 7, 8, Supplementary Figures S1, S2, and Supplementary Tables S4, S5 show that DMSC can generate the most robust estimations.

### Experiment 4: V4 Variable Region Dataset From the Murine Gut

In this experiment, we adopt another real-world benchmark dataset of the V4 variable region from the Murine gut to assess the performance of OTUs picking methods. The V4 dataset was generated by Illumina’s MiSeq platform (Westcott and Schloss, 2015), covering the V4 hypervariable region of 16S rRNAs from Murine microbiota [36]. The raw sequences of V4 dataset can be freely obtained from http:/www.mothur.org/MiSeqDevelopmentData/StabilityNoMetaG.tar. The ground-truth of V4 dataset can be extracted as followings. First, the pair end raw sequences were merged by FLASH (Magoč and Salzberg, 2011), then the usearch (Edgar, 2010) program was adopted to filter the merged sequences. Finally, the Python script (assign_taxonomy.py) in QIIME (Caporaso et al., 2010) was used to align the sequences for obtaining the ground-truth information with a stringent criterion. If the identity percentage is more than 97% (≥97%) and the length of the aligned region is more than 90% (≥90%) of the total length, the annotated sequences are retained. Thus, we obtained about ∼511K annotated reads, which were classified into 68 genera.

**FIGURE 9:**
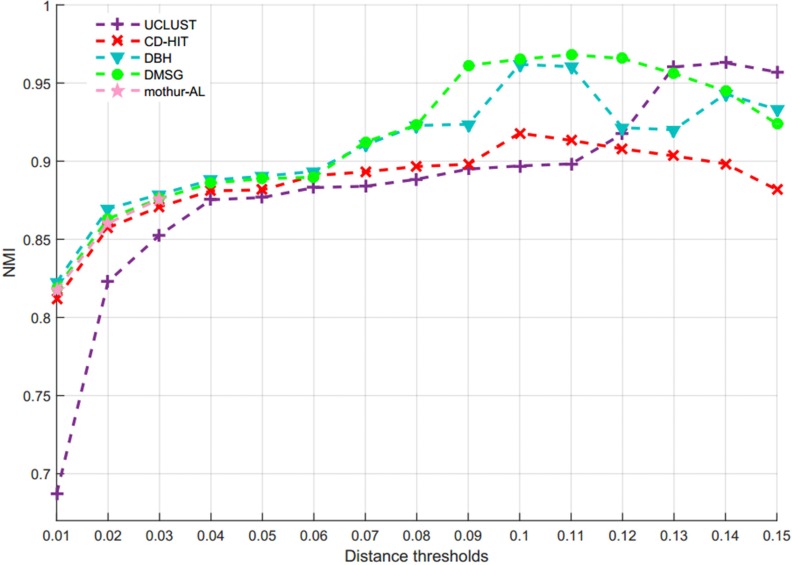
NMI values of five methods at different distance thresholds on V4 dataset.

**FIGURE 10:**
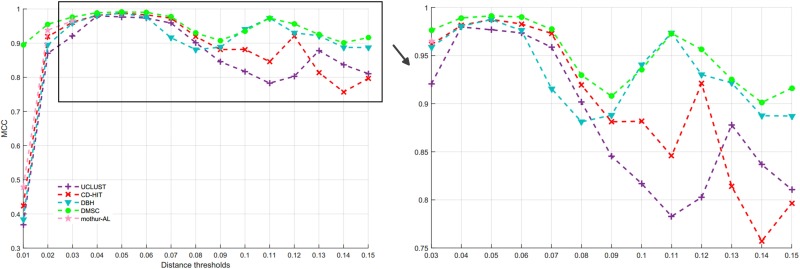
MCC values of five methods at different distance thresholds on V4 dataset.

By setting different distance thresholds ranging from 0.01 to 0.15, the NMI curves of five methods are shown in Figure 9, and the inferred OTUs number of five methods at different distance threshold are presented in Supplementary Figure S3. Figure 10 is the MCC curves of five methods at different distance thresholds. The NMI values, OTUs number and MCC values inferred with five methods at different distance thresholds are listed in Supplementary Table S6. Because DySC software returns a debug information, ESPRIT-Forest appears a segmentation fault (core dumped) information, and CROP is time-consuming on this large V4 dataset, we did not give the results of DySC, ESPRIT-Forest, and CROP in this experiment.

From Figure 9, we can see that most of NMI values of DMSC are higher than that of other four methods in the range of 0.01∼0.13 distance thresholds, and it is obviously higher than other three methods in the distance range of 0.09∼0.12. The results in Supplementary Figure S3 show that DMSC and DBH inferred less OTUs than other methods, and DMSC inferred 67 OTUs which is near the ground truth at 0.09 distance threshold. From Figure 10, we can see that the MCC values of DMSC are higher than that of the other four methods except at 0.10 distance threshold. These results suggest that DMSC can achieve higher clustering quality than UCLUST, CD-HIT, DBH, and mothur-AL methods.

## Discussion

Inspired by the seed reselection strategy and model-based methods, we herein developed a novel dynamic multi-seeds heuristic method for picking OTUs from 16S rRNA sequences. Besides the distance threshold *θ* given by users, DMSC also needs another two parameters in picking OTUs procedure: η and μ. How these two parameters affect the clustering results needs to be further investigated. In the following, we tested the parameter effect on the simulated dataset used in experiment 2. We first tested the effect of the η by fixing μ (e.g., μ = 3). The NMI values at different distance thresholds are presented in Supplementary Figure S4, from which we can see that we can see that the NMI values of η = 10, 15, 20, 15 in the range of 0.02∼0.1 distance thresholds are nearly equal, indicating that η has little influence on the clustering results. Supplementary Figure S5 shows the effect of μ by fixing η (e.g., η = 25). From Supplementary Figure S5, we found that the NMI values of μ = 3, 4 are higher than that of μ = 1, 2 in the range of 0.01∼0.1 distance thresholds. Therefore, we select η = 25 and μ = 3 as the default parameter values in our DMSC method.

Sequencing errors (i.e., deletion, insertion, and substitution) are inevitably introduced during the high-throughput sequencing procedure, which can easily lead to OTUs overestimation (Schmidt et al., 2015). In order to estimate the robustness of handling sequencing errors for different OTU picking methods, ten simulated datasets in DBH (Wei and Zhang, 2017) with error rate varies from 0.21 to 0.42% are used to test our DMSC method. Each dataset contains 150,000 sequences from 30 taxa and each taxon contains 5,000 sequences. The OTUs number inferred at 0.05 distance threshold is shown in Supplementary Figure S6, from which we can see that with the error rate increase from 0.21 to 0.41%, DMSC infer a smaller number of OTUs than other methods, especially in the 0.33 ∼ 0.41% scope of higher error rate, the OTUs number inferred by DMSC is obviously less than that of other five methods. Table 4 lists the average OTUs number and std (σ) in the scope of 0.21∼0.41% sequencing errors, from which we can see that the average OTUs number of DMSC is smaller than that of other five methods, and the standard deviation is lower than that of UCLUST, DBH, CD-HIT, and ESPRIT-Forest, near to DySC. Supplementary Table S7 reports the average OTUs number and std at 0.03 distance threshold, from which we can see that the standard deviation of DMSC is lower than that of other five methods. These results indicate that DMSC can better reduce the OTUs overestimation than the other five methods.

**Table 4:** Average OTUs number and standard deviation of six methods in the scope of 0.21∼0.41% sequencing errors at 0.05 distance threshold.

	DMSC	UCLUST	DBH	CD-HIT	DySC	ESPRIT-Forest
Average OTUs	34	38	37	39	46	92
σ	3.748	9.605	6.863	11.253	3.588	24.691


The rapid increase in the amount of sequencing data provides a valuable source to significantly understand bacterial diversity from the environmental samples, meanwhile introducing a serious computational challenge for processing these mass data. In addition to the clustering accuracy, computational complexity is also used to assess a new clustering method. The computational complexity of DMSC mainly contains three components. (1) For generating clusters, a total of *N* sequences needs to be processed. The large maximum complexity is *O*(*N*). (2) In the MCS selection procedure, a distance matrix with size of η × η needs to be calculated with a complexity of *O*(*K* × η^2^), where *K* is the number of clusters with size larger than η. (3) In the sequences assignment procedure, each sequence is compared with each cluster, resulting in a complexity of *O*(*K* × *N*). As a result, the total time complexity of DMSC is O(*N*+*K* ×η^2^+*K* × *N*), which is larger than that of traditional heuristic clustering methods such as CD-HIT and UCLUST, but smaller than that of model-based clustering methods such as CROP. In this work, all methods were executed with 16 threads. In order to graphically demonstrate the scaling property of our DMSC method, we compared DMSC with CD-HIT, UCLUST, DBH, DySC, mothur-AL and ESPRIT-Tree on V6 dataset at different sequence size ranging from 1 K to 100 M. Supplementary Figure S7 shows the running time (wall time) of seven methods. We can see that with the sequence number increases, the speed of DMSC is much faster than mothur-AL, and little lower than the traditional heuristic methods (e.g., CD-HIT, UCLUST, and DBH) that just use one sequence as the seed for each cluster. For the memory usage, Supplementary Figure S8 graphically describes the memory property of seven methods. From Supplementary Figure S8, we can see that DMSC needs a little larger memory usage than the classical greedy clustering methods such as CD-HIT, UCLUST and DySC, and much smaller memory storage than ESPRIT-Forest and mothur-AL for large-scale sequences.

## Conclusion

16S rRNA high-throughput sequencing has become a powerful and convenient technology for studying microbial diversity and composition in the environmental samples. Until now, numerous heuristic clustering methods have been developed to pick OTUs, but most of them just select one sequence as the cluster seed, resulting in OTUs overestimation and sensitivity to the sequencing errors. In this work, we proposed a novel dynamic multi-seeds heuristic clustering method (namely DMSC) by incorporating the dynamical multi-seeds updating strategy and the heuristic clustering procedure. Meanwhile, DMSC considers the distance’s standard deviation within the MCS to generate OTUs. DMSC method is inspired by the idea of seed reselection procedure in DySC, but there are three main differences between DMSC and DySC: (i) DMSC selects MCS as the seeds in one cluster, while DySC just uses one single sequence as the seed; (ii) DySC only updates seed once time, then the seed will be fixed, while DMSC dynamically updates the MCS if a new sequence is added to one cluster, therefore, the seeds is always updated with the cluster size increases; and (iii) a new sequence is assigned to the corresponding cluster depending on the average distance to MCS and the distance standard deviation between each pairwise sequences in MCS, while DySC assigns the new sequence just based on the distance to seed sequence. Compared with the state-of-the-art methods, such as UCLUST, CD-HIT, DBH, DySC, ESPRIT-Forest, CROP, and mothur-AL, the clustering results show that DMSC can produce OTUs with higher quality and reduce OTUs overestimation with low memory usage. Additionally, DMSC is also robust to the sequencing errors.

## Data Availability

The DMSC software is available at https://github.com/NWPU-903PR/DMSC, the datasets used and/or analyzed during the current study are available from the corresponding references or from the corresponding author on reasonable request.

## Author Contributions

Z-GW wrote the code and manuscript, and developed the software. S-WZ designed the study and revised the manuscript. Both authors contributed to the conception and design of the study, participated in the data analysis, and to writing and editing of the manuscript. Both authors read, edited, and approved the final manuscript.

## Conflict of Interest Statement

The authors declare that the research was conducted in the absence of any commercial or financial relationships that could be construed as a potential conflict of interest.

## Abbreviations

AL: average linkage
MCC: matthews correlation coefficient
MCS: multi-core sequences
OTU: operational taxonomic units
rRNA: ribosomal RNA
std: standard deviations
