# Supplementary material for: DMSC: A Dynamic Multi-Seeds Method for Clustering 16S rRNA Sequences Into OTUs
Source: Front Microbiol. 2019 Mar 12;10:428. doi: 10.3389/fmicb.2019.00428 (PMC6422886; doi:10.3389/fmicb.2019.00428)
Supplement: Supplementary file 1 [file Presentation_1.pdf]

Supplementary files

DMSC: a dynamic multi-seeds method for clustering 16S rRNA sequences into OTUs

Ze-Gang Wei[1,2], Shao-Wu Zhang[1]*

1 Key Laboratory of Information Fusion Technology of Ministry of Education, School of Automation, Northwestern Polytechnical University, Xi'an 710072, China

2 Institute of Physics and Optoelectronics Technology, Baoji University of Arts and Science, Baoji 721016, China

* Corresponding author. Email: zhangsw@nwpu.edu.cn

A. NMI and MCC calculation

In this study, we applied NMI (normalized mutual information) and MCC (Matthew's correlation coefficient) metrics to evaluate the clustering performance of different methods. Here we show the computational formulas of NMI and MCC.

NMI is commonly used to evaluate the clustering accuracy, that is, how the outcome of one clustering algorithm agree with the ground truth [1]. Suppose that there are N reads from M species $\Omega=\{s_1,s_2,...,s_M\}$ and they are clustered into C OTUs $\Pi=\{o_1,o_2,...,o_C\}$ at a specific distance threshold by a clustering algorithm. The NMI is calculated by:

$$\begin{cases} NMI(\Omega,\Pi)=\dfrac{2I(\Omega,\Pi)}{H(\Omega)+H(\Pi)} \\ I(\Omega,\Pi)=\sum_{i=1}^{M}\sum_{j=1}^{C}\dfrac{a_{ij}}{N}\log\dfrac{a_{ij}/N}{|s_i||o_j|/N^2} \\ H(\Omega)=-\sum_{i=1}^{S}\dfrac{s_i}{N}\log\dfrac{s_i}{N} \\ H(\Pi)=-\sum_{j=1}^{C}\dfrac{o_j}{N}\log\dfrac{o_j}{N} \end{cases} \tag{1}$$

where $I(\Omega,\Pi)$ is the mutual information and $H(\Pi)$ is the entropy. $|s_i|$ denotes the number of reads in s_i, $|o_j|$ denotes the number of reads in o_j, and a_{ij} represents the number of reads from s_i and categorized into o_j. NMI has a value range [0, 1] and

larger NMI value implies better clustering accuracy.

MCC calculation is based on the sequence distance and clustering threshold without relying on the ground truth, which is an objective metric to assess the clustering quality of OTUs picking methods [2]. For a set of sequences assigned to OTUs at a distance threshold clustered by one clustering algorithm, we counted the number of sequence pairs that could be considered as true positives (TPs), true negatives (TNs), false positives (FPs), and false negatives (FNs). A pair of sequences was considered as a TP if the distance between the sequences was smaller than the distance threshold and they belonged to the same OTU; a FP was a pair of sequences that belonged to the same OTU but had a pairwise distance larger than the threshold. A pair of sequences was considered as a true negative (TN) if their pairwise distance was larger than the threshold and they did not belong to the same OTU; a false negative (FN) was a pair of sequences that belonged to different OTUs, but had a pairwise distance smaller than the threshold. The MCC is defined by:

$$MCC = \frac{TP \times TN - FP \times FN}{\sqrt{(TP+FP)(TP+FN)(TN+FP)(TN+FN)}} \quad (2)$$

The MCC value can vary between -1 and 1 and larger MCC value represents better clustering quality.

[1] Chen W, Cheng Y, Zhang C, et al. MSClust: a multi-seeds based clustering algorithm for microbiome profiling using 16S rRNA sequence. Journal of microbiological methods, 2013, 94(3): 347-355.

[2] Schloss P D, Westcott S L. Assessing and improving methods used in OTU-based approaches for 16S rRNA gene sequence analysis. Applied and environmental microbiology, 2011.

B. Supplementary Tables

Table S1. Running command lines of different clustering methods.

Programs	Running command lines
DBH	db -i seq.fasta -c 0.97 -o dbh97 -T 16
DySC	DySC-v0 -in seq.fasta -th 0.97 -out dysc97
CROP	CROPLunix -i seq.fasta -o crop97 -s
DMSC	dmsc -i seq.fasta -o dmsc97.txt -c 0.97 -T 16
CD-HIT	cd-hit -i seq.fasta -c 0.97 -o cdhit97 -T 16 -M 0
ESPRIT-Forest	ESFclust seq.fasta
UCLUST	usearch -sortbylength seq.fasta -fastaout sorted.fasta
	usearch -cluster_fast sorted.fasta -id 0.97 -uc uclust97.uc
mothur-AL	unique.seqs(fasta=seq.fasta)
	dist.seqs(fasta=unique.fa, cutoff=0.11, processors=16)
	cluster(column=unique.dist, name=seq.names, method=average, cutoff=0.10)
	bin.seqs(list=seq.unique.an.list, fasta=seq.fasta)

Table S2. The NMI, MCC values and OTUs number of eight methods at different distance thresholds on Stacked_60 dataset.

Distance	DMSC			UCLUST			CD-HIT			DBH		
	NMI	MCC	OTUs	NMI	MCC	OTUs	NMI	MCC	OTUs	NMI	MCC	OTUs
0.01	0.69547	0.3498	2212	0.69486	0.2173	2300	0.80345	0.2702	1160	0.83392	0.2620	1040
0.02	0.88640	0.9042	626	0.81373	0.6962	942	0.97932	0.7545	125	**0.99868**	0.7781	62
0.03	**0.99951**	**0.9999**	59	0.93127	0.8273	205	0.99075	0.9817	84	0.99861	**0.9985**	59
0.04	0.99951	0.9999	59	0.95838	0.8970	136	**0.99557**	**0.9910**	65	0.99655	0.9921	59
0.05	0.99906	0.9988	59	0.97112	0.9260	104	0.99529	0.9882	59	0.99424	0.9774	59
0.06	0.99348	0.9848	59	0.97429	0.9240	96	0.99330	0.9775	58	0.99057	0.9626	57
0.07	0.99075	0.9913	56	0.97238	0.8968	92	0.98892	0.9849	58	0.98686	0.9766	55
0.08	0.98986	0.9987	56	0.97784	0.9060	82	0.98411	0.9799	57	0.98598	0.9839	55
0.09	0.98683	0.9919	53	**0.98528**	**0.9348**	56	0.98155	0.9738	54	0.98183	0.9725	52
0.10	0.98233	0.9899	46	0.98417	0.9150	56	0.97597	0.9599	54	0.97878	0.9778	51

Table S2-1. The NMI, MCC values and OTUs number of eight methods at different distance thresholds on Stacked_60 dataset.

Distance	DySC			ESPRIT-Forest			CROP			mothur-AL		
	NMI	MCC	OTUs	NMI	MCC	OTUs	NMI	MCC	OTUs	NMI	MCC	OTUs
0.01	0.81507	0.2683	1152	0.73966	0.1946	1507	0.93702	0.1947	338	0.68787	0.2080	2429
0.02	0.99460	0.7780	79	0.83160	0.6884	724	0.99913	0.7762	60	0.82750	0.6214	910
0.03	**0.99475**	**0.9847**	60	0.94390	0.8478	142	**0.99951**	**0.9999**	59	0.89922	0.7961	463
0.04	0.98978	0.9554	59	0.95512	0.8779	119	0.99951	0.9999	59	0.94049	0.8833	266
0.05	0.98660	0.9433	56	0.95485	0.8682	113	0.99951	0.9989	59	0.95945	0.9201	189
0.06	0.98100	0.9332	56	0.95800	0.8806	104	0.98947	0.9605	57	**0.96650**	**0.9234**	161
0.07	0.96645	0.8815	54	0.96282	0.9102	94	0.98617	0.9751	55	-	-	-
0.08	0.95028	0.8315	53	**0.96614**	**0.9373**	86	0.97460	0.9527	55	-	-	-
0.09	0.94829	0.8060	78	0.96544	0.9150	56	0.93865	0.7949	53	-	-	-
0.10	0.93256	0.7290	70	0.95833	0.8752	54	0.89180	0.6341	46	-	-	-

mothur-AL software just outputs the clustering results at distance thresholds from 0.01 to 0.06.

Table S3. The NMI, MCC values and OTUs number of seven methods at different distance thresholds on simulated dataset.

Distance	DMSC			UCLUST			CD-HIT			DBH			DySC			ESPRIT-Forest			CROP		
	NMI	MCC	OTUs	NMI	MCC	OTUs	NMI	MCC	OTUs	NMI	MCC	OTUs	NMI	MCC	OTUs	NMI	MCC	OTUs	NMI	MCC	OTUs
0.01	0.9334	0.8808	99	0.7252	**0.9369**	528	0.5991	0.8295	927	0.7777	0.7080	439	0.6243	0.8940	941	0.5930	0.8903	1198	0.9197	0.5842	158
0.02	**0.9503**	0.9287	9	0.7759	0.8536	97	0.7160	0.8979	159	0.9161	0.8818	17	0.8140	0.9096	174	0.7815	0.9249	299	0.9320	0.8818	9
0.03	0.9434	0.9879	9	0.8524	0.7997	26	0.8555	**0.9840**	27	**0.9293**	**0.9868**	9	**0.9252**	**0.9838**	17	0.9114	0.9677	46	**0.9334**	0.9868	9
0.04	0.9307	**0.9980**	9	0.9000	0.8903	13	0.9267	0.9780	11	0.9106	0.9739	9	0.8950	0.9555	10	0.8967	**0.9947**	16	0.9333	**0.9980**	9
0.05	0.9223	0.9585	8	**0.9107**	0.9046	10	**0.9334**	0.8657	9	0.8433	0.8526	8	0.8641	0.9323	8	**0.8979**	0.9568	13	0.9157	0.9485	9
0.06	0.8958	0.9218	6	0.8814	0.8319	9	0.9009	0.8835	9	0.8298	0.8345	8	0.8497	0.8622	7	0.8529	0.8762	12	0.8526	0.8477	7
0.07	0.8698	0.9370	6	0.8552	0.7842	8	0.8518	0.9205	8	0.8054	0.8522	7	0.7700	0.7754	6	0.8512	0.8868	11	0.7986	0.9066	6
0.08	0.8353	0.9323	6	0.8169	0.7676	7	0.8244	0.9000	8	0.7986	0.9417	6	0.6874	0.5617	5	0.7956	0.9412	10	0.7784	0.9185	6
0.09	0.8261	0.9086	5	0.8174	0.7335	7	0.8052	0.9306	7	0.7866	0.9411	6	0.6365	0.5498	5	0.7445	0.9406	7	0.6301	0.5078	5
0.10	0.7966	0.9100	5	0.7898	0.6958	7	0.7652	0.9309	7	0.7686	0.9073	6	0.6107	0.5052	5	0.7245	0.9073	7	0.5185	0.7673	4

Table S4. The NMI, MCC values and OTUs number of six methods at different distance thresholds on V6 dataset.

Distance	DMSC			UCLUST			CD-HIT			DBH			DySC			ESPRIT-Forest		
	NMI	MCC	OTUs	NMI	MCC	OTUs	NMI	MCC	OTUs	NMI	MCC	OTUs	NMI	MCC	OTUs	NMI	MCC	OTUs
0.01	0.5978	0.4940	4775	0.5851	0.5081	5342	0.5661	0.3720	5001	0.5954	0.3879	4987	0.5918	0.2325	3352	0.5684	0.2064	5686
0.02	0.6521	0.4450	2422	0.6419	0.4169	2943	0.6088	0.3777	2809	0.6402	0.3988	2417	0.6249	0.2108	1299	0.6146	0.3129	3292
0.03	0.7266	0.4071	1320	0.6887	0.3746	1790	0.6401	0.3637	1748	0.7222	0.4010	1306	0.7067	0.2240	576	0.6727	0.3454	1651
0.04	0.8070	0.4066	744	0.7330	0.3532	1000	0.6762	0.3812	1092	0.7966	0.4260	710	0.7900	0.3162	257	0.7518	0.3657	853
0.05	0.8801	0.5420	340	0.8047	0.3715	481	0.7180	0.4628	659	0.8771	0.5832	319	0.8589	0.5514	155	0.8252	0.4197	458
0.06	0.9294	0.7144	208	0.8376	0.4256	340	0.7478	0.5874	466	0.9143	0.7077	208	0.8904	0.6444	137	0.9042	0.5648	274
0.07	**0.9487**	0.8384	160	0.8666	0.4549	256	0.7719	0.6832	373	0.9356	0.8190	155	**0.8945**	0.7235	117	0.9290	0.7179	192
0.08	0.9362	0.8729	123	0.8858	0.5134	201	0.7937	0.7956	301	0.9436	0.8760	129	0.8803	**0.7529**	99	0.9331	0.7559	156
0.09	0.9235	0.8852	107	0.9151	0.5647	169	0.8048	**0.8277**	250	**0.9437**	0.9225	115	0.8671	0.6125	76	0.9440	0.7840	127
0.10	0.9006	**0.9389**	94	**0.9382**	**0.6190**	132	**0.8275**	0.8112	214	0.9318	**0.9340**	98	0.8033	0.5373	62	**0.9456**	**0.9288**	115

Table S5. The *P*-values of the Student's t-test between DMSC and other methods on the V6 dataset.

	UCLUST	CD-HIT	DBH	DySC	ESPRIT-Forest
DMSC	3.21E-15	5.62E-07	0.0738	5.38E-08	0.5165

Table S6. The NMI, MCC values and OTUs number of six methods at different distance thresholds on the V4 dataset.

Distance	DMSC			UCLUST			CD-HIT			DBH			mothur-AL		
	NMI	MCC	OTUs	NMI	MCC	OTUs	NMI	MCC	OTUs	NMI	MCC	OTUs	NMI	MCC	OTUs
0.01	0.8187	0.8955	4378	0.6871	0.3681	11576	0.8117	0.4235	4839	0.8225	0.3844	4035	0.8173	0.4788	4037
0.02	0.8632	0.9552	852	0.8229	0.8702	1044	0.8574	0.9189	806	0.8695	0.8946	613	0.8603	0.9384	806
0.03	0.8760	0.9765	326	0.8526	0.9205	448	0.8707	0.9614	353	0.8787	0.9587	240	**0.8757**	**0.9649**	275
0.04	0.8859	0.9891	169	0.8752	**0.9797**	197	0.8810	0.9814	181	0.8879	0.9806	142	-	-	-
0.05	0.8888	**0.9913**	133	0.8767	0.9768	159	0.8816	**0.9876**	145	0.8902	**0.9875**	126	-	-	-
0.06	0.8897	0.9902	115	0.8830	0.9736	119	0.8907	0.9828	120	0.8934	0.9761	99	-	-	-
0.07	0.9123	0.9773	95	0.8839	0.9587	108	0.8931	0.9731	102	0.9103	0.9157	90	-	-	-
0.08	0.9231	0.9297	78	0.8884	0.9015	95	0.8966	0.9194	88	0.9228	0.8811	77	-	-	-
0.09	0.9612	0.9078	67	0.8949	0.8454	82	0.8979	0.8812	80	0.9235	0.8881	72	-	-	-
0.10	0.9651	0.9355	61	0.8969	0.8170	70	**0.9179**	0.8817	67	**0.9618**	0.9406	60	-	-	-
0.11	**0.9681**	0.9731	58	0.8982	0.7829	62	0.9133	0.8457	61	0.9604	0.9735	52	-	-	-
0.12	0.9656	0.9563	48	0.9175	0.8027	56	0.9078	0.9210	55	0.9214	0.9302	47	-	-	-
0.13	0.9560	0.9252	44	0.9602	0.8780	49	0.9033	0.8141	49	0.9201	0.9216	43	-	-	-
0.14	0.9448	0.9012	40	**0.9629**	0.8367	45	0.8983	0.7572	44	0.9430	0.8875	38	-	-	-
0.15	0.9239	0.9162	35	0.9570	0.8108	43	0.8817	0.7965	43	0.9331	0.8872	37	-	-	-

mothur-AL software just outputs the clustering results at 0.01, 0.02 and 0.03 distance thresholds.

Table S7. Average OTUs number and standard deviation of six methods in the scope of 0.21%~0.41% sequencing errors at 0.03 distance threshold.

	DMSC	UCLUST	DBH	CD-HIT	DySC	ESPRIT-Forest
Average OTUs	214	238	209	560	238	271
σ	98.43	108.55	103.26	318.55	106.36	147.39

C. Supplementary Figures

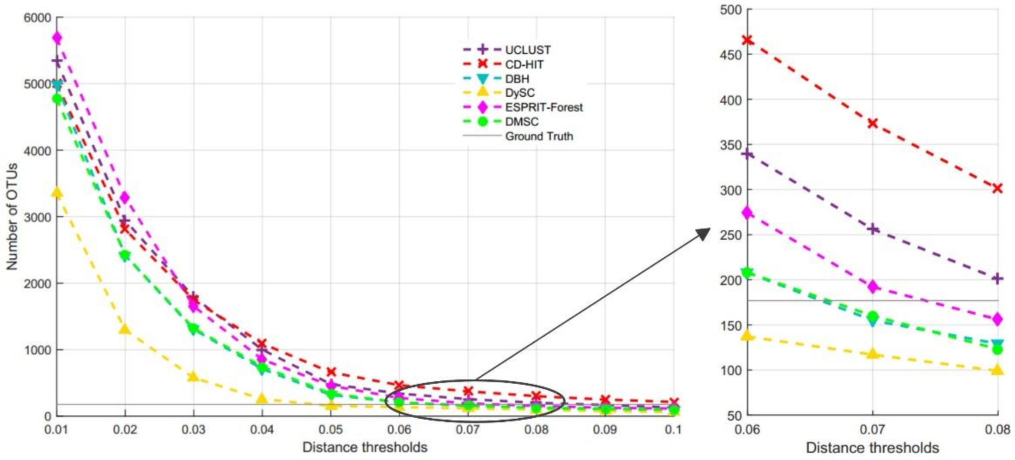

Figure S1. The average OTU number inferred with six methods at different distance thresholds on V6 dataset.

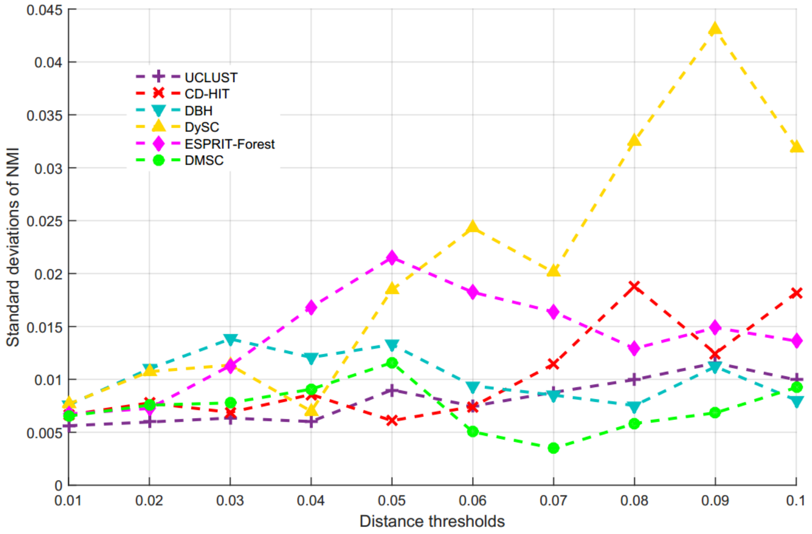

Figure S2. Standard deviations of NMI for six methods with 10 runs on V6 dataset.

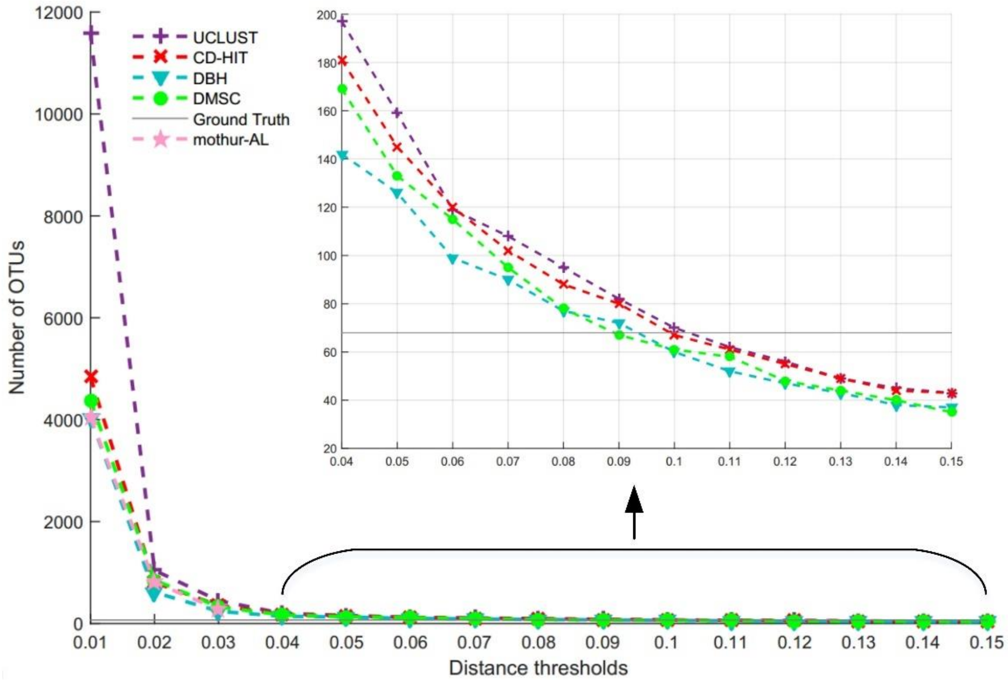

Figure S3. OTU number inferred with five methods at different distance thresholds on V4 dataset.

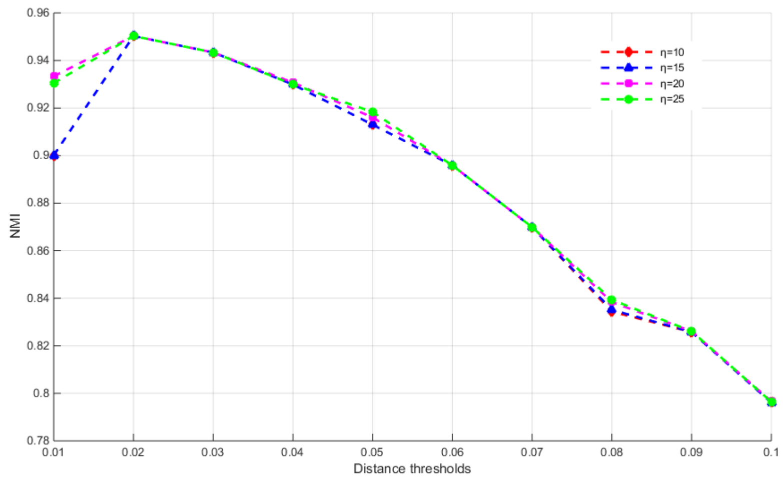

Figure S4. Influence of the parameter η by fixing μ=3.

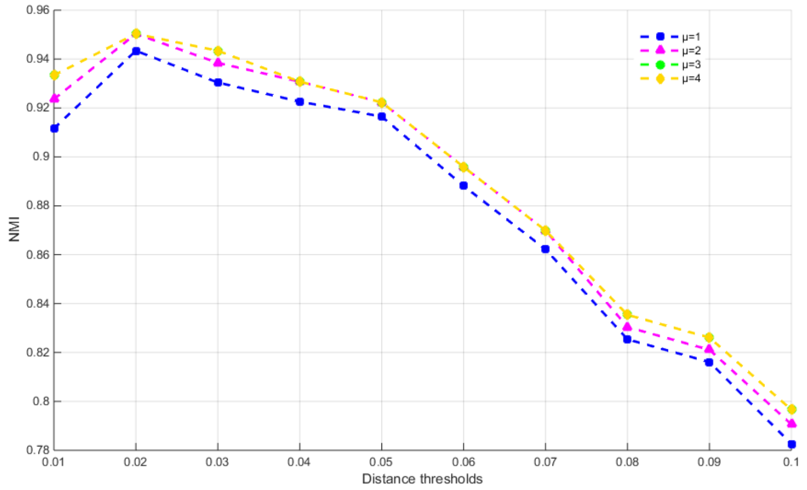

Figure S5. Influence of the parameter μ by fixing η=25.

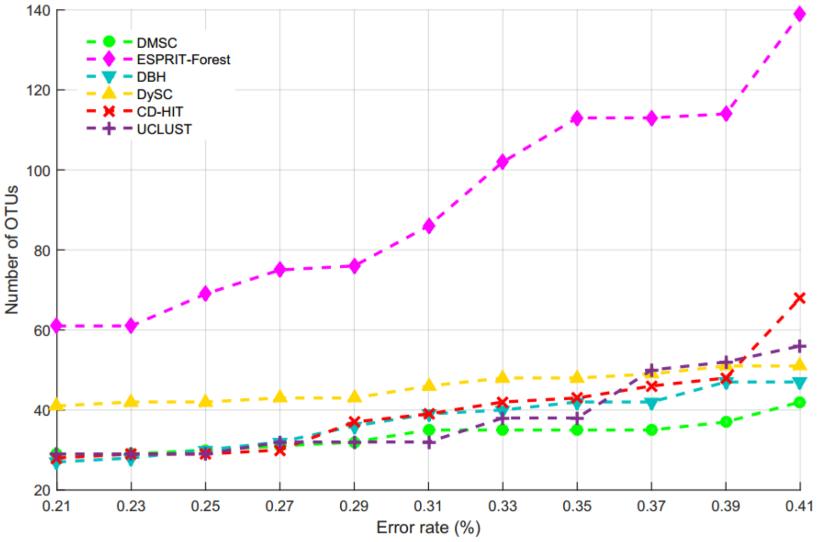

Figure S6. The OTU number inferred with six algorithms on a simulated dataset with different error rates.

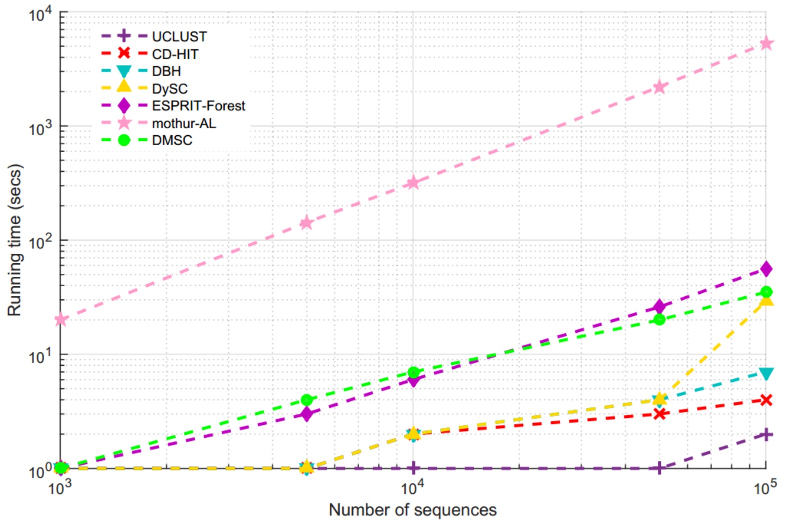

Figure S7. Running time of DMSC, CD-HIT, UCLUST, DHB, DySC, ESPRIT-Forest and mothur-AL on the V6 dataset with sequence number ranging from 1 K to 100 K.

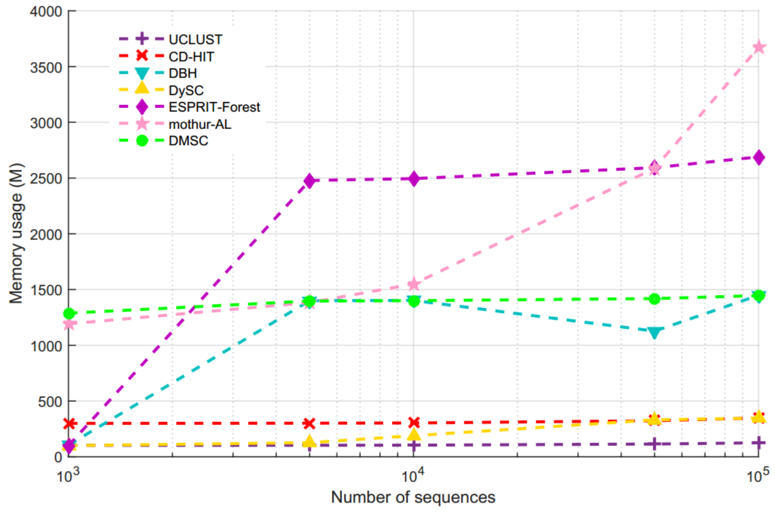

Figure S8. Memory usage of seven methods on V6 dataset with sequences ranging from 1 K to 100 K.
